# Neutrophil-to-Lymphocyte Ratio Is Associated with the Stability of Human Corneal Endothelial Cells

**DOI:** 10.3390/jcm15072538

**Published:** 2026-03-26

**Authors:** Gyeong Min Lee, Ye Eun Lee, Young Joo Shin

**Affiliations:** 1 Department of Ophthalmology, Hallym University Medical Center, Hallym University College of Medicine, Seoul 07441, Republic of Korea; 2 Hallym BioEyeTech Research Center, Hallym University College of Medicine, Seoul 07441, Republic of Korea

**Keywords:** corneal endothelial cells, neutrophil-to-lymphocyte ratio, specular microscopy

## Abstract

**Background/Objectives**: Corneal endothelial cells (CEnCs) exist on the inner surface of the cornea and regulate its hydration. The immune system cannot penetrate CEnCs easily because the cornea is avascular and anterior chamber-associated immune deviation suppresses the immune reaction. Nevertheless, inflammatory cells can infiltrate through the corneal stroma and anterior chambers, and corneal endothelial inflammation can occur. In this cross-sectional study, we investigated the association between the neutrophil-to-lymphocyte ratio (NLR) and human corneal endothelial cells (CEnCs). **Methods**: A total of 307 eyes from 307 subjects who underwent specular microscopy were included. Corneal endothelial cell density (CECD), hexagonality (HEX), central corneal thickness (CCT), coefficient of variation (CV), and cell area were measured preoperatively using specular microscopy. Whole blood samples were obtained to measure the complete blood cell count. The NLR was calculated, and its relationship with CEnCs was evaluated. **Results**: In all subjects, CV was positively correlated with the percentage (%) of neutrophils (r = 0.120, *p* = 0.037) and absolute neutrophil count (r = 0.131, *p* = 0.022) and negatively correlated with the % of lymphocytes (r = −0.131, *p* = 0.022). HEX was correlated with the % of neutrophils (r = −0.156, *p* = 0.006), % of lymphocytes (r = 0.141, *p* = 0.014), % of basophils (r = 0.142, *p* = 0.013), the NLR (r = −0.129, *p* = 0.024), and the mean corpuscular volume (r = 0.121, *p* = 0.035). **Conclusions**: CV and HEX, which indicate the stability of CEnCs, are associated with NLR in the peripheral blood, suggesting that systemic inflammation and immunity may implicate in the pathology of CEnCs.

## 1. Introduction

The cornea, a transparent avascular tissue forming the anterior surface of the eye, plays a pivotal role in maintaining visual clarity by providing approximately two-thirds of the eye’s refractive power and serving as a protective barrier against external insults. Among the three main layers of the cornea—epithelium, stroma, and endothelium—the corneal endothelial cells (CEnCs) occupy a uniquely critical position on the innermost surface, directly interfacing with the aqueous humor of the anterior chamber [1,2]. These highly specialized cells are essential for maintaining corneal transparency through their role in regulating stromal hydration and preventing corneal edema [3,4]. CEnCs form a monolayer of predominantly hexagonal cells that function as a metabolically active pump-leak system [1,2,4]. Despite their continuous metabolic activity, including high levels of adenosine triphosphate (ATP) production and active ion transport mechanisms involving Na^+^/K^+^-ATPase pumps, CEnCs exhibit remarkable stability and longevity under physiological conditions [3,4]. Unlike corneal epithelial cells, which continuously regenerate throughout life, human CEnCs are arrested in the G1 phase of the cell cycle and do not undergo mitotic division under normal circumstances [1,2]. This growth-arrested state, established during early postnatal development, means that any loss of CEnCs due to injury, disease, or aging cannot be compensated by cellular proliferation [1,2]. Instead, the remaining cells respond by undergoing cellular migration and hypertrophy, increasing their individual surface area to maintain coverage of the posterior corneal surface [1,5]. This compensatory mechanism has significant clinical implications: while the cornea can tolerate gradual CEnC loss to a certain degree, there exists a critical threshold below which endothelial function becomes compromised, leading to corneal decompensation [6].

CEnCs are evaluated by the cell shape and density in two dimensions [7], with specular microscopy being a widely used method [8,9]. Although CEnCs increase the cell area and cover the posterior area of the cornea in response to injury or disease, a decrease in the number of CEnCs below 400–500 cells/mm^2^ leads to difficulty in cataract surgery and may result in bullous keratopathy, which requires corneal transplantation [10,11]. The risk factors for reduced density or function of CEnCs include heredity [12], trauma [13,14], systemic factors (such as drugs) [15,16], aging [17], diabetes [18], smoking [19], and vitamin D deficiency [20]. These diseases have been reported to be associated with oxidative stress and the dysregulation of immune system.

The cornea benefits from a unique immunological status known as immune privilege, which protects it from many inflammatory [21,22]. Despite these protective mechanisms, the immune privilege of the cornea is not absolute [23]. Under certain pathological conditions, the barriers can be breached, allowing inflammatory cells to infiltrate through the corneal stroma and anterior chamber, ultimately reaching the corneal endothelium. Such breaches occur prominently in corneal graft rejection following transplantation, where donor CEnCs become targets of host immune responses mediated by both cellular and humoral immunity, and viral infections, particularly herpes simplex virus (HSV) and varicella-zoster virus (VZV), can cause herpes endotheliitis—a condition characterized by localized or diffuse inflammation of the corneal endothelium [24,25].

Systemic inflammation is increasingly recognized as a contributor to microvascular and cellular dysfunction in various tissues. Neutrophils are the first responders against external pathogens, removing them by degranulation, phagocytosis, cytokine production, and neutrophil extracellular trap formation (NETosis) [26,27]. Neutrophils are the effector cells of the innate immune system, while lymphocytes carry out the adaptive immune response [27]. As such, the neutrophil-to-lymphocyte ratio (NLR), a readily available biomarker derived from routine blood counts, reflects the balance between innate and adaptive immune responses and has been associated with cardiovascular disease, metabolic disorders, and other inflammatory conditions [28,29]. High NLR is associated with inflammatory diseases and vascular diseases [30,31,32,33,34] because neutrophils and NETosis regulate the adaptive immunity [35]. Therefore, we hypothesized that systemic inflammation, as reflected by NLR, is associated with morphologic instability of corneal endothelial cells. The purpose of this study was to investigate the association between peripheral blood inflammatory markers and corneal endothelial cell parameters in patients undergoing routine cataract surgery.

## 2. Materials and Methods

### 2.1. Study Design and Ethical Considerations

This study employed a retrospective, cross-sectional design to investigate the relationship between systemic inflammatory markers and corneal endothelial cell parameters in patients undergoing routine cataract surgery. This retrospective study was performed in accordance with the tenets of the Declaration of Helsinki and was reviewed and approved by the institutional review board/ethics committee of Hallym University Medical Center. This waiver was justified on the grounds that (1) the research posed no more than minimal risk to participants; (2) the waiver would not adversely affect the rights and welfare of the subjects; (3) the research could not practicably be carried out without the waiver; and (4) appropriate safeguards were implemented to protect patient confidentiality and data security.

### 2.2. Study Setting and Population

This study was conducted at Hallym University Kangnam Sacred Heart Hospital, a tertiary academic medical center located in Seoul, South Korea. Medical records were reviewed retrospectively for all patients who underwent cataract surgery at our institution during the period from November 2019 to January 2021. Patients with corneal opacity, corneal dystrophy, keratoconus, or a history of previous intraocular surgery or intraocular inflammation were excluded. Patients with active systemic inflammatory, autoimmune, infectious, glaucoma, pseudoexfoliation syndrome, or malignant diseases at the time of surgery or history of systemic and topical medications that could influence endothelial status were excluded based on medical record review. Common chronic conditions such as diabetes mellitus and hypertension were not used as exclusion criteria in order to reflect a real-world cataract population. All corneal endothelial measurements were obtained preoperatively. All cataract surgeries were performed using standardized techniques by experienced surgeons at our institution. This study did not include intraoperative parameters or postoperative endothelial outcomes, which limits assessment of surgical impact and clinical risk prediction. Only the right eye of the patients was included in this study. To avoid statistical complications arising from inter-eye correlation in bilateral data and to maintain independence of observations—a fundamental assumption of many statistical tests—only data from the right eye of each patient were included in the analysis. This approach is consistent with methodological recommendations for ophthalmic research and has been widely employed in previous epidemiological studies of corneal endothelium. In cases where only left eye data were available due to technical factors (e.g., poor image quality in the right eye), those patients were excluded from the study to maintain consistency in laterality.

### 2.3. Corneal Endothelial Cell Assessment

All specular microscopy measurements were performed preoperatively before cataract surgery. Although cataract grade was not available, endothelial parameters were measured preoperatively; therefore, they were not affected by phacoemulsification energy or surgical time. Corneal endothelial cell morphology and density were evaluated using non-contact specular microscopy (Topcon SP-2000P; Topcon Corporation, Tokyo, Japan). This instrument utilizes a non-contact optical system based on the principle of specular reflection from the corneal endothelial cell layer, capturing high-resolution images without requiring direct contact with the cornea, thereby eliminating risks of corneal abrasion, infection, or patient discomfort. The captured specular microscopy images were analyzed using the instrument’s built-in automated image analysis software, which employs sophisticated algorithms for cell border recognition and morphometric quantification. The center method was used for cell identification, wherein the software identifies cell centers and constructs boundaries equidistant from adjacent centers. All images were obtained by trained technicians using a standardized protocol. Images with poor focus or indistinct cell borders were excluded, and only the best-quality image with at least 100 contiguous cells were selected. Automated analysis using the instrument’s built-in software was applied in all cases. A minimum of 100 contiguous cells were analyzed per image, with the best quality image selected for each patient. Corneal endothelial cell density (CECD), coefficient of variation (CV), hexagonality (HEX), central corneal thickness (CCT), and cell area were measured using specular microscopy (Topcon SP-2000P; Topcon Corporation, Tokyo, Japan).

### 2.4. Hematologic Analysis

Peripheral venous blood samples were obtained from all patients as part of the routine preoperative laboratory evaluation required for surgical clearance. Blood was collected into EDTA (ethylenediaminetetraacetic acid) anticoagulant tubes (typically 4 mL lavender-top tubes) for complete blood count analysis. EDTA is the anticoagulant of choice for hematology testing as it preserves cell morphology, prevents platelet clumping, and provides stable cell counts for several hours when stored properly. Tubes were gently inverted 8–10 times immediately after collection to ensure thorough mixing with the anticoagulant. Samples were labeled with patient identifiers, date, time of collection, and phlebotomist initials. Samples were transported to the hospital laboratory within 1 h of collection and maintained at room temperature (not refrigerated) to preserve cell integrity. All hematologic analyses were performed in the Hallym University Kangnam Sacred Heart Hospital Clinical Laboratory, which maintains accreditation from the Korean Association of Quality Assurance for Clinical Laboratories (KAQACL) and participates in external quality assurance programs to ensure analytical accuracy and reliability. Whole blood samples were obtained for hematologic analysis using a hematology analyzer (Siemens ADVIA 2120i; Siemens Healthcare Diagnostics, Erlangen, Germany), and the following were analyzed: white blood cell (WBC) count with absolute neutrophil count, percentage (%) of neutrophils, lymphocytes, monocytes, eosinophils, and basophils; red blood cell (RBC) count with mean corpuscular volume (MCV), mean corpuscular hemoglobin (MCH), mean corpuscular hemoglobin concentration, hemoglobin (Hb), hematocrit (Hct), and RBC distribution width; and platelet count with mean platelet volume, plateletcrit, and platelet distribution width. The NLR was also calculated.

### 2.5. Data Collection and Management

Demographic, clinical, and laboratory data were extracted from the hospital’s electronic medical record (EMR) system by trained research personnel. A standardized data collection form was used to ensure consistency and completeness. The correlation of the parameters of CEnCs from specular microscopy and each blood cell count were evaluated in all subjects and according to sex.

#### Statistics

The Statistical Package for the Social Sciences 27.0 software (IBM Inc., Armonk, NY, USA) was used for statistical analysis. Correlations between measurements for CEnCs and each blood cell count were evaluated using Pearson’s correlation analysis. The Pearson’s correlation coefficient r was calculated, and a *p*-value less than 0.05 was considered statistically significant. An independent *t*-test was used for comparison between the two groups. Eyes with baseline CECD < 1500 cells/mm^2^ were excluded from additional sensitivity analyses. Multivariable linear regression analyses were performed to evaluate the independent association between systemic inflammatory markers and corneal endothelial parameters after adjusting for age, sex, diabetes, hypertension, and CECD. Multivariable linear regression analyses were performed with CV and HEX as dependent variables and NLR, age, sex, diabetes, hypertension, CCT and CECD as independent variables.

## 3. Results

### 3.1. Baseline Characteristics

A total of 307 eyes from 307 subjects (139 men and 168 women) were included in the analyses. The mean age was 68.19 ± 11.16 years. The demographic data are shown in Table 1. The mean values of the variables measured from specular microscopy were: CECD, 2609.50 ± 337.38 cells/mm^2^; HEX, 55.87 ± 14.48%; cell area, 389.00 ± 56.56 μm^2^; CV, 34.27 ± 8.21%; and CCT, 515.52 ± 35.65 μm. Associations between HEX or CV and age or CCT were shown in Supplementary Figure S1.

### 3.2. Association of Corneal Endothelial Cells and Hematologic Analysis

**Figure 1:**
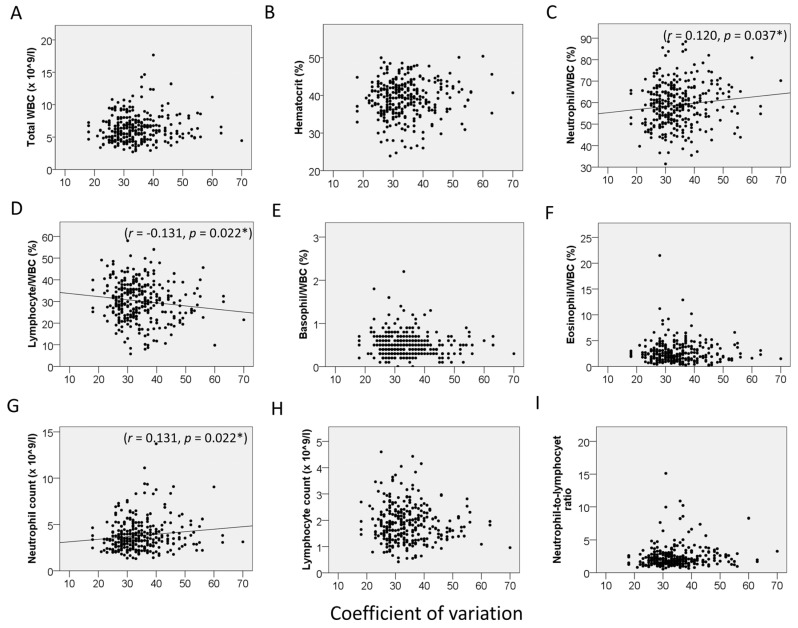
Correlation between coefficient of variation and hematologic parameters. (**A**) Total white blood cell (WBC) count. (**B**) Hematocrit. (**C**) Neutrophil/WBC ratio. (**D**) Lymphocyte/WBC ratio. (**E**) Basophil/WBC ratio. (**F**) Eosinophil/WBC ratio. (**G**) Absolute neutrophil count. (**H**) Absolute lymphocyte count. (**I**) Neutrophil-to-lymphocyte ratio. * *p* < 0.05 indicates statistical significance.

**Figure 2:**
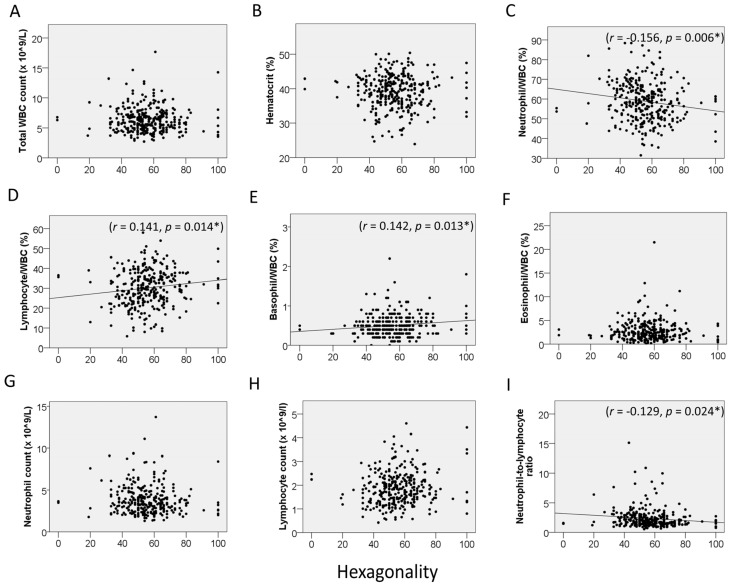
Correlation between hexagonality and hematologic parameters. (**A**) Total white blood cell (WBC) count. (**B**) Hematocrit. (**C**) Neutrophil/WBC ratio. (**D**) Lymphocyte/WBC ratio. (**E**) Basophil/WBC ratio. (**F**) Eosinophil/WBC ratio. (**G**) Absolute neutrophil count. (**H**) Absolute lymphocyte count. (**I**) Neutrophil-to-lymphocyte ratio. * *p* < 0.05 indicates statistical significance.

On hematologic analysis, the following were noted: mean WBC count, 6.3429 ± 2.1051 × 10^9^/L; RBC count, 4.2353 ± 0.5597 × 10^9^/L; Hb, 13.15 ± 1.72 g/dL; Hct, 39.22 ± 4.87%; MCV, 92.88 ± 5.54 fL; MCH, 31.15 ± 1.99 pg; platelet, 226.58 ± 65.80 × 10^9^/L; neutrophil, 58.88 ± 10.00%; lymphocyte, 30.18 ± 8.97%; monocyte, 5.66 ± 2.60%; eosinophil, 2.68 ± 2.12%; basophil, 0.50 ± 0.27%; delta neutrophil index, 0.0607 ± 0.3053; neutrophil count, 3.80 ± 1.70 × 10^9^/L; lymphocyte count, 1.86 ± 0.70 × 10^9^/L; monocyte count, 0.35 ± 0.15 × 10^9^/L; eosinophil count, 0.17 ± 0.15 × 10^9^/L; basophil count, 0.031 ± 0.017 × 10^9^/L; and NLR, 2.35 ± 1.65. The relationship between CEnCs and blood cells was also investigated. In all subjects, CV was positively correlated with the % of neutrophils (r = 0.120, *p* = 0.037) and absolute neutrophil count (r = 0.131, *p* = 0.022) and negatively correlated with the % of lymphocytes (r = −0.131, *p* = 0.022) (Figure 1). HEX was correlated with the % of neutrophils (r = −0.156, *p* = 0.006), % of lymphocytes (r = 0.141, *p* = 0.014), % of basophils (r = 0.142, *p* = 0.013), the NLR (r = −0.129, *p* = 0.024), and MCV (r = 0.121, *p* = 0.035) (Figure 2).

**Figure 3:**
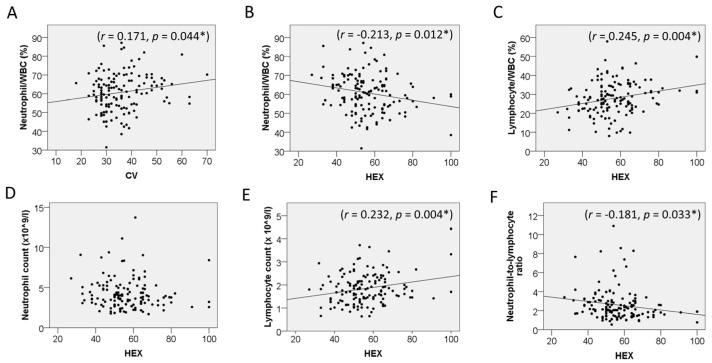
Correlation between corneal endothelial cells and blood cells in male. (**A**) Correlation between coefficient of variation (CV) and neutrophil/WBC ratio. (**B**) Correlation between hexagonality (HEX) and neutrophil/WBC ratio. (**C**) Correlation between HEX and lymphocyte/WBC ratio. (**D**) Correlation between HEX and absolute neutrophil count. (**E**) Correlation between HEX and absolute lymphocyte count. (**F**) Correlation between HEX and neutrophil-to-lymphocyte ratio. CV = coefficient of variation; HEX = hexagonality. * *p* < 0.05 indicates statistical significance.

**Figure 4:**
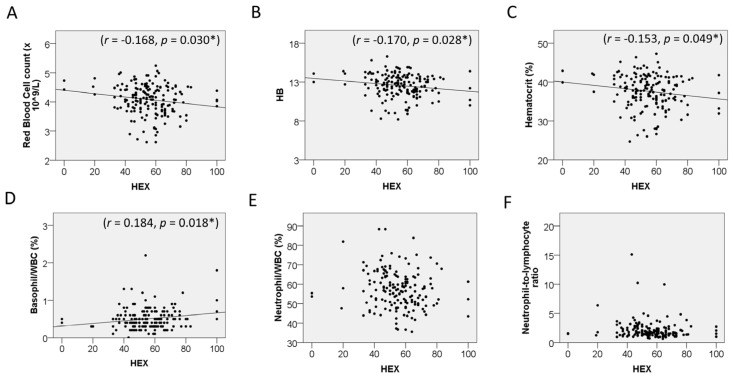
Correlation between corneal endothelial cells and blood cells in female. (**A**) Red blood cell count. (**B**) Hemoglobin (Hb). (**C**) Hematocrit. (**D**) Basophil/WBC ratio. (**E**) Neutrophil/WBC ratio. (**F**) Neutrophil-to-lymphocyte ratio. HEX = hexagonality. * *p* < 0.05.

The groups were also divided by sex to compare the relationship between CEnCs and blood cells. In males, CV was positively correlated with the % of neutrophils (r = 0.171, *p* = 0.044), and HEX was correlated with the % of neutrophils (r = −0.213, *p* = 0.012), % of lymphocytes (r = 0.245, *p* = 0.004), and the NLR (r = −0.181, *p* = 0.033) (Figure 3). In females, HEX was correlated with the RBC count (r = −0.168, *p* = 0.030), Hb (r = −0.170, *p* = 0.028), Hct (r = −0.153, *p* = 0.049), and the % of basophil (r = 0.184, *p* = 0.018) (Figure 4).

Multivariable linear regression analysis showed that NLR remained significantly associated with HEX (*p* = 0.030), after exclusion of eyes with CECD < 1500 cells/mm^2^ and adjustment for age, sex, diabetes, hypertension, CCT and CECD.

## 4. Discussion

This retrospective cross-sectional study investigated the relationship between systemic inflammatory markers, particularly the neutrophil-to-lymphocyte ratio (NLR), and morphometric parameters of corneal endothelial cells (CEnCs) assessed by specular microscopy in patients undergoing routine cataract surgery. To our knowledge, this is the first study to systematically examine the association between peripheral blood inflammatory markers and corneal endothelial health in a clinical population without overt ocular inflammation.

Multiple factors are associated with the number and functions of CEnCs [36]. The relationship between systemic inflammation and CEnCs has not been reported. Our principal findings demonstrate that markers of endothelial cell stability—specifically the coefficient of variation (CV) and hexagonality (HEX)—are significantly correlated with systemic inflammatory markers. In addition, this study also revealed that CECD, cell size, and CCT were correlated with age, which is in accordance with previous studies [37,38]. CV, the ratio of the standard deviation to the mean area, and HEX, the % of hexagonal cells, indicate CEnC stability [39]. A low CV and a high HEX value indicate a stable cornea with good function [39]. When damaged, the CEnCs heal the injury through cellular hypertrophy, resulting in high CV and low HEX [40]. This study revealed that the CV and HEX are associated with NLR. NLR, which reflects the dynamic relationship between innate (neutrophils) and adaptive cellular immune response (lymphocytes), is an inflammatory state marker that predicts the prognosis of cardiovascular disease [41,42] and diabetic retinopathy [43]. Excessive neutrophil levels are associated with NETosis [44], which is implicated in inflammation [45]. Although the anterior chamber is an immune privilege site inaccessible to the immune system [46] and clinical signs are invisible, the subclinical inflammation and NETosis may persistently affect the corneal endothelium. CEnCs secrete the neutrophil chemotactic factor [47], with neutrophils being present in keratic precipitates [48,49] and the transplanted cornea [50]. CEnCs express adhesion molecules that bind to the receptors on the surface of neutrophils or macrophages [51,52]. These findings suggest that neutrophils have an effect on CEnCs.

When divided by sex, the relationship between NLR and CEnCs was statistically significant in men but not in women. This difference may be due to the younger age of men and lower WBC count in women. Furthermore, men have various risk factors, such as smoking and exposure to air pollutants, which are known to increase NLR [53]. Another reason could be the effect of hormones. In women, the production of estrogen, which affects the NLR [54], is accelerated after menopause. Meanwhile, in men, the androgen (testosterone) levels show only a gradual decline with age. Neutrophil counts in women fluctuate during the menstrual cycle and the increased % of neutrophils are associated with serum estradiol [55,56]. Testosterone administration in men differentially increases neutrophil and monocyte counts [57]. In this study, the changes in CEnCs in females were associated with Hb or Hct in the peripheral blood. The association between CV or HEX and anemia has been reported [58]. Oxygen supply is essential to CEnCs because these are metabolically active [3,4]. Low oxygen supply due to anemia may cause injury to CEnCs. Furthermore, anemia induces neutrophil hypersegmentation [59] and neutropenia [60]. Local inflammatory markers were not measured; therefore, the study cannot determine whether NLR represents a causal mechanism or a surrogate marker of systemic inflammation.

Several mechanisms may explain the association between NLR and corneal endothelial morphologic instability. Low-grade systemic inflammation may promote subclinical anterior chamber inflammation, allowing circulating inflammatory mediators to influence the corneal endothelium despite ocular immune privilege. Proinflammatory cytokines such as tumor necrosis factor-α and interleukin-6 have been shown to impair Na^+^/K^+^-ATPase activity and mitochondrial function, potentially compromising endothelial pump efficiency and altering cell morphology. In addition, elevated NLR has been associated with increased oxidative stress, which can damage cytoskeletal proteins and intercellular junctions, leading to increased pleomorphism and reduced hexagonality.

In multivariable models, the standardized effect size of NLR on CV and HEX was smaller than that of age but comparable to that of CCT, suggesting that systemic inflammatory status may represent a modest yet independent contributor to corneal endothelial vulnerability. These findings support the concept that systemic health influences corneal endothelial stability even in the absence of clinically apparent ocular inflammation.

This study has several limitations. Its retrospective, cross-sectional, single-center design precludes causal inference and may limit generalizability. Standardized cataract grading, intraoperative parameters, postoperative endothelial outcomes, and formal inter- and intra-observer reproducibility analyses were not included, limiting assessment of surgical relevance and measurement reliability. Prospective longitudinal studies with standardized systemic and ocular assessments are warranted.

## 5. Conclusions

In conclusion, CV and HEX, which indicate the stability of CEnCs, are associated with NLR in the peripheral blood. These results suggest that systemic inflammation and immunity may be implicated in the pathology of CEnCs.

## Figures and Tables

**Table 1:** Demographic characteristics.

	Total	Male	Female	*p*-Value
**General information**				
N	307	139	168	
Age (y)	68.19 ± 11.16	66.05 ± 11.86	69.96 ± 10.24	0.002 *
**Corneal endothelial cell parameter**				
CECD (cells/mm^2^)	2609.50 ± 337.38	2617.72± 348.35	2602.74± 328.98	0.700
HEX (%)	55.87 ± 14.48	56.12 ± 13.13	55.66 ± 15.54	0.785
Cell area (μm^2^)	389.00 ± 56.56	387.09 ± 62.29	390.58 ± 51.47	0.591
CV (%)	34.27 ± 8.21	36.00 ± 9.18	32.84 ± 7.03	0.001 *
CCT (μm)	240.41 ± 258.46	242.43 ± 260.36	238.73 ± 257.64	0.901
**Complete blood cell count**				
WBC (×10^9^/L)	6.34 ± 2.11	6.876 ± 2.25	5.896 ± 1.87	<0.001 *
RBC (×10^9^/L)	4.23 ± 0.56	4.42 ± 0.56	4.08 ± 0.51	<0.001 *
Hct (%)	39.22 ± 4.87	41.14 ± 4.81	37.61 ± 4.32	<0.001 *
Hb (g/dL)	13.15 ± 1.72	13.88 ± 1.70	12.54 ± 1.50	<0.001 *
MCV (femtoliter)	92.88 ± 5.54	93.52 ± 6.58	92.33 ± 4.43	0.063
Neutrophil (%)	58.88 ± 10.00	60.78 ± 10.09	57.29 ± 9.67	0.002 *
Lymphocyte (%)	30.18 ± 8.97	27.84 ± 8.70	32.13 ± 8.75	<0.001 *
Monocyte (%)	5.66 ± 2.60	5.71 ± 1.56	5.61 ± 3.23	0.723
Eosinophil (%)	2.68 ± 2.12	2.93 ± 1.97	2.46 ± 2.22	0.057
Basophil (%)	0.50 ± 0.27	0.504 ± 0.246	0.505 ± 0.292	0.970
DNI	0.061 ± 0.31	0.063 ± 0.298	0.058 ± 0.312	0.889
Neutrophil count (×10^9^/L)	3.80 ± 1.70	4.25 ± 1.92	3.42 ± 1.40	<0.001 *
Lymphocyte count (×10^9^/L)	1.86 ± 0.70	1.85 ± 0.67	1.87 ± 0.73	0.820
Monocyte count (×10^9^/L)	0.35 ± 0.15	0.38 ± 0.14	0.31 ± 0.14	<0.001 *
Eosinophil count (×10^9^/L)	0.17 ± 0.15	0.20 ± 0.16	0.14 ± 0.13	0.001 *
Basophil count (×10^9^/L)	0.031 ± 0.017	0.033 ± 0.018	0.028 ± 0.015	0.006 *
NLR	2.35 ± 1.65	2.61 ± 1.64	2.13 ± 1.63	0.011 *

* statistically significant by independent *t*-test. CECD = corneal endothelial cell density; HEX = hexagonality; CV = coefficient of variation; CCT = central corneal thickness; WBC = white blood cell; RBC = red blood cell; Hct = hematocrit; MCV = mean corpuscular volume; DNI = Delta neutrophil index; NLR = neutrophil-to-lymphocyte ratio.

## Data Availability

The data supporting the findings of this study are available from the corresponding author upon reasonable request.

## Supplementary Materials

The following supporting information can be downloaded at: https://www.mdpi.com/article/10.3390/jcm15072538/s1.

## Abbreviations

The following abbreviations are used in this manuscript:

CEnC	corneal endothelial cells
CECD	corneal endothelial cell density
HEX	hexagonalit
CCT	central corneal thickness
CV	coefficient of variation
NLR	neutrophil-to-lymphocyte ratio
