# Supplementary figures and images for: Neutrophil-to-Lymphocyte Ratio Is Associated with the Stability of Human Corneal Endothelial Cells
Source: J Clin Med. 2026 Mar 26;15(7):2538. doi: 10.3390/jcm15072538 (PMC13073400; doi:10.3390/jcm15072538)

Supplementary Figure S1.

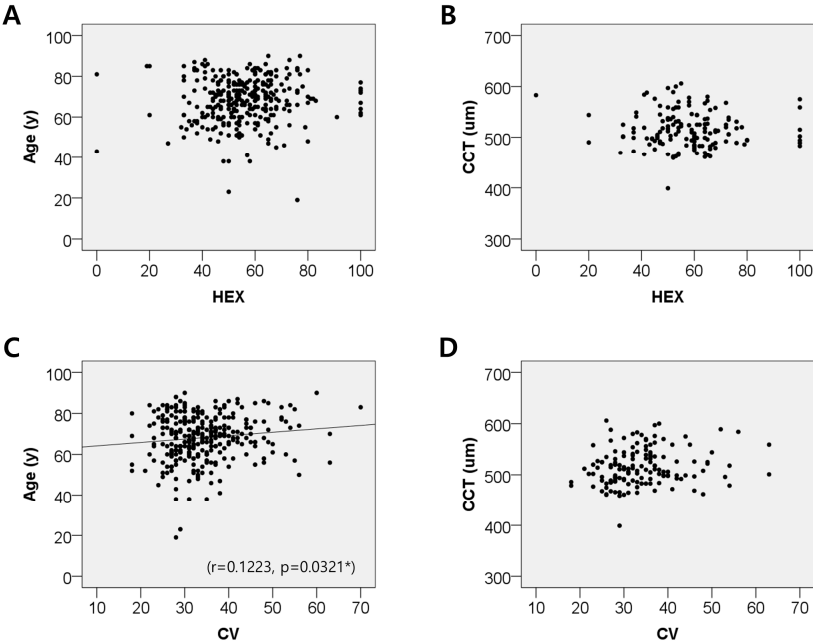

Supplement: Supplementary file 1 [file jcm-15-02538-s001.zip › jcm-4152295-supplementary.pdf]
